# Chlorine-Infused Wide-Band Gap p-CuSCN/n-GaN Heterojunction Ultraviolet-Light Photodetectors

**DOI:** 10.1021/acsami.1c22075

**Published:** 2022-04-11

**Authors:** Jian-Wei Liang, Yuliar Firdaus, Chun Hong Kang, Jung-Wook Min, Jung-Hong Min, Redha H. Al Ibrahim, Nimer Wehbe, Mohamed Nejib Hedhili, Dimitrios Kaltsas, Leonidas Tsetseris, Sergei Lopatin, Shuiqin Zheng, Tien Khee Ng, Thomas D. Anthopoulos, Boon S. Ooi

**Affiliations:** † Photonics Laboratory, Computer, Electrical and Mathematical Sciences and Engineering Division (CEMSE), King Abdullah University of Science and Technology (KAUST), Thuwal 23955-6900, Saudi Arabia; ‡ Physical Science and Engineering Division (PSE), KAUST Solar Center (KSC), King Abdullah University of Science and Technology (KAUST), Thuwal 23955-6900, Saudi Arabia; § Imaging and Characterization Core Labs, King Abdullah University of Science and Technology (KAUST), Thuwal 23955-6900, Saudi Arabia; ∥ Currently with Research Center for Electronics and Telecommunication, National Research and Innovation Agency, Jalan Sangkuriang Komplek LIPI Building 20 Level 4, Bandung 40135, Indonesia; ⊥ Department of Physics, School of Applied Mathematical and Physical Sciences, National Technical University of Athens, Athens GR-15780, Greece

**Keywords:** copper thiocyanate, gallium nitride, ultraviolet-based photodetector, X-ray photoelectron spectroscopy, p-CuSCN/n-GaN heterojunction photodetector

## Abstract

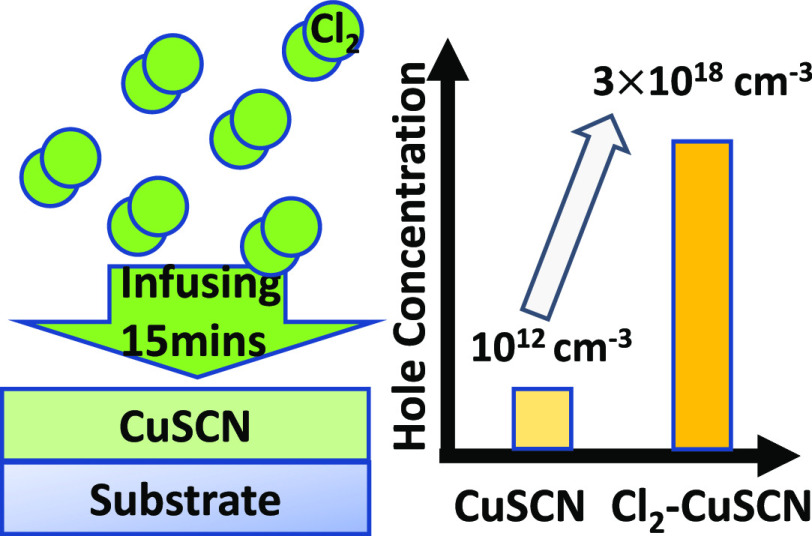

Copper thiocyanate (CuSCN) is a p-type semiconductor that exhibits hole-transport and wide-band gap (∼3.9 eV) characteristics. However, the conductivity of CuSCN is not sufficiently high, which limits its potential application in optoelectronic devices. Herein, CuSCN thin films were exposed to chlorine using a dry etching system to enhance their electrical properties, yielding a maximum hole concentration of 3 × 10^18^ cm^–3^. The p-type CuSCN layer was then deposited onto an n-type gallium nitride (GaN) layer to form a prototypical ultraviolet-based photodetector. X-ray photoelectron spectroscopy further demonstrated the interface electronic structures of the heterojunction, confirming a favorable alignment for holes and electrons transport. The ensuing p-CuSCN/n-GaN heterojunction photodetector exhibited a turn-on voltage of 2.3 V, a responsivity of 1.35 A/W at −1 V, and an external quantum efficiency of 5.14 × 10^2^% under illumination with ultraviolet light (peak wavelength of 330 nm). The work opens a new pathway for making a plethora of hybrid optoelectronic devices of inorganic and organic nature by using p-type CuSCN as the hole injection layer.

## Introduction

The lack of efficient p-type materials represents bottlenecks in wide- and ultra-wide band gap semiconductor technologies. For instance, ultraviolet optoelectronics based on aluminum gallium nitride (AlGaN) suffer from poor external quantum efficiency (EQE) due to the low hole concentration and low conductivity of p-type aluminum gallium nitride (p-AlGaN). Even worse, the higher the composition of Al in p-AlGaN, the lower the hole concentration and conductivity.^1,2^ This issue not only increases the device resistance but also causes difficulty in forming an ohmic contact with the metal. Mitigation of this conductivity disadvantage could significantly increase the potential of AlGaN for deep UV optoelectronics. Several methods have been reported to increase the hole concentration in the p-type III-nitride layer. In simulations, Mg delta-doping of an AlN/GaN superlattice led to a lower acceptor activation energy and increased the hole concentration.^3,4^ Another method to surmount this difficulty is to grow p-type GaN (p-GaN) on p-AlGaN directly and then grow less than 5 nm heavily doped p-GaN (p^++^-GaN) at the very end of the p-GaN layer to reduce the contact resistance between the metal contact and the p-type layer. This p^++^-GaN/p-GaN/p-AlGaN sandwich structure is widely utilized in ultraviolet-based light-emitting diodes (LEDs) and laser diodes (LDs).^5−8^ Even though these reports argue that Mg delta-doped p-GaN layers have a higher hole concentration, the p-GaN layers will absorb UV light, which reduces the EQE and therefore significantly impacts the performance of the corresponding UV-based optoelectronic devices. Hence, p-type materials that possess a higher hole concentration with a wider band gap than p-GaN (3.4 eV) have been desperately sought after for decades.

Copper thiocyanate (CuSCN) offers several desirable features that hold the potential to improve III-nitride-based devices. For example, its indirect band gap is 3.9 eV with a relatively low effective mass of holes.^9^ These unique properties make CuSCN an ideal hole-transporting layer^10^ in organic devices, such as organic photovoltaics,^11−15^ hybrid perovskite solar cells,^16−19^ and organic light-emitting diodes.^20,21^ Moreover, CuSCN can also be used as an absorbing layer in UV optoelectronic devices, for example, metal–semiconductor–metal (M–S–M) ultraviolet-based photodetectors (UVPDs),^22,23^ and as a p-type layer in p–n junction UVPDs.^24^

In addition to its electronic properties, CuSCN also has exceptionally versatile processing properties. Various solution-processing methods can be utilized to deposit CuSCN at low temperatures, including spin-coating,^11,20,25^ ink-jet printing,^26,27^ doctor blading,^28,29^ and electrochemical deposition.^30−32^ These advantages enable the growth of CuSCN films with thicknesses in the range from 10 to several 100 s of nanometers, which facilitates the application of CuSCN in p-channel thin-film transistors (p-TFTs).^33,34^ Solvents, such as diethyl sulfide (DES) and dipropyl sulfide, are commonly used to dissolve CuSCN. Due to the inert, stable, and noncorrosive nature of these solvents, the resulting solutions do not damage commonly used substrate materials such as SiO_2_, Al_2_O_3_, and indium tin oxide, during solution processing. Additionally, DES and dipropyl sulfide do not dissolve most organic molecules, making CuSCN ideal for processing on top of the active layers.^16,35^

Despite its promising characteristics, the low hole concentration and conductivity of CuSCN limit the gamut of its potential applications. Even though it has been argued that the p-type dopant concentration of CuSCN is in the order of 7.2 × 10^17^ cm^–3^,^36^ its low doping efficiency leads to poor conductivity at room temperature. Exposure to halogen gases or post-treatment with salt thiocyanate are the methods generally used to increase the conductivity of CuSCN layers.^37−39^ Both approaches were found to yield SCN^–^ ions and increase the carrier concentration in CuSCN layers.^40^ Exposure to chlorine (Cl_2_) has been reported to be the most efficient of these doping approaches.^37^ To explore Cl_2_ doping of CuSCN under stable conditions, we used a dry etching system to expose solution-processed CuSCN thin films to Cl_2_ gas under controlled conditions, that is, background pressure and Cl_2_ concentration.

Here, we report chlorine-infused CuSCN thin films that exhibit the properties of a wide-band gap p-type material with a maximum hole concentration of 3 × 10^18^ cm^–3^. The chlorine-infused CuSCN thin films were characterized by complementary techniques. A Cl_2_-doped CuSCN layer was then combined with n-GaN to produce a prototype p-CuSCN/n-GaN heterojunction UVPD with excellent operating characteristics. This photodetector exhibited a turn-on voltage of 2.3 V, a maximum responsivity of 1.35 A/W at an applied bias of −1 V, and an EQE of 5.14 × 10^2^% under illumination by UV light with a peak wavelength of 330 nm. This work paves the way toward innovative CuSCN-based inorganic semiconductor devices and sheds light on the possibilities of new classes of optoelectronics based on p-type CuSCN.

## Experimental Section

### Epitaxial Growth of n-GaN Using Molecular Beam Epitaxy and Fabrication of a p-CuSCN/n-GaN Ultraviolet-Based Photodetector

A 200 nm n-GaN layer was grown on an undoped-GaN/sapphire template with a 7 μm undoped GaN layer by VEECO GEN930 plasma-assisted molecular beam epitaxy (MBE). For the growth conditions, the cell temperatures of silicon and gallium were 1180 and 898 °C, respectively, and the substrate temperature was 700 °C. The nitrogen plasma was operated at a nitrogen flow rate of 0.6 SCCM with a power of 250 W. Supporting Information S1 provides details of the fabrication of the p-CuSCN/n-GaN heterojunction UVPD.

### Characterization of the p-CuSCN/n-GaN Ultraviolet-Light Photodetector

The electrical characterization of the p-CuSCN/n-GaN UVPD was performed using a customized measuring station, which consists of an arc lamp as a light source, an optical setup guiding the light to the fabricated device, and a semiconductor parametric analyzer to measure the optoelectronic characteristics (Agilent Technologies 4156B). Neutral density (ND) filters were adopted to manipulate the output power from the arc lamp to calibrate the light intensity.

### Secondary Ion Mass Spectrometry

Dynamic secondary ion mass spectrometry (SIMS) experiments were performed using a Hiden instrument (Warrington, UK) operated under ultra-high vacuum conditions (typically 10^–9^ Torr). A continuous Ar^+^ beam was employed at 4 keV to sputter the sample surface, while the selected ions were sequentially collected using a MAXIM spectrometer equipped with a quadrupole analyzer. The raster of the sputtered area was approximately 750 × 750 μm^2^. To avoid the edge effect during the depth profiling process, data were recorded from a smaller area located in the middle of the sputtered region. The acquisition area was adjusted using adequate electronic gating to about 75 × 75 μm^2^.

### X-ray Photoelectron Spectrometry

X-ray photoelectron spectroscopy (XPS) studies were carried out using a Kratos Axis Supra DLD spectrometer equipped with a monochromatic Al Kα X-ray source (*h*ν = 1486.6 eV) operating at 45 W, a multichannel plate, and delay line detector under a vacuum of ∼10^–9^ mbar. All spectra were recorded using an aperture slot of 300 × 700 μm. Survey spectra were collected using a pass energy of 160 eV and a step size of 1 eV. A pass energy of 20 eV and a step size of 0.1 eV were used for the high-resolution spectra. For XPS analysis, the samples were mounted in floating mode to avoid differential charging. Charge neutralization was required for all samples. Binding energies were referenced to the C 1s binding energy of adventitious carbon contamination, which was taken to be 284.8 eV.

### Time Response Measurements

The measurements were performed using a customized optical setup that included a 266 nm laser, a probe manipulator, a viewing camera, a beam chopper, and a sample holder. The signals were detected using a digital storage oscilloscope (DSOX1102G, Keysight). The 266 nm laser was generated by third harmonic generation through a two-step process involving two sequential harmonic generation steps (800 nm + 800 nm → 400 nm, 800 nm + 400 nm → 266 nm); the whole setup was pumped by an 800 nm, 3.5 W, 120 fs and 76 MHz mode-locked laser. The final power of the 266 nm laser was about 200 mW.

### Density Functional Theory Calculations

The calculations employed the density functional theory (DFT) code Vienna Ab initio Simulation Package,^41^ utilizing a plane-wave basis (with an energy cutoff of 400 eV), projector augmented waves,^42^ and the generalized gradient approximation Perdew–Burke–Ernzerhof^43^ exchange–correlation (xc) functional. Sampling of the reciprocal space used the Monkhorst-Pack^44^ scheme (tetrahedron method)^45^ in total energy [density of states, (DOS)] calculations. As in previous DFT studies^46^ on the bulk and defect properties of CuSCN, we used large supercells with a total of 128 atoms in the pristine case. Structures were rendered with the software VESTA.^47^

## Results and Discussion

**Figure 1:**
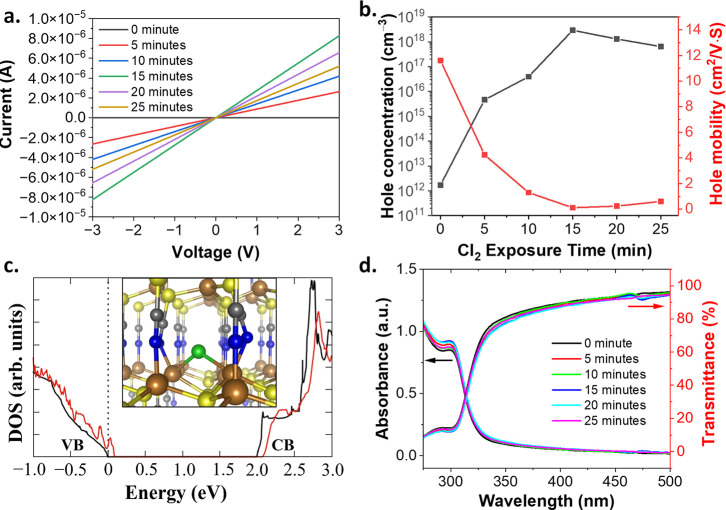
Optical and electrical characterization of the intrinsic CuSCN and the Cl_2_-infused CuSCN thin films, and DFT simulation of Cl_2_-infused CuSCN: (a) *I*–*V* curves of CuSCN thin films exposed to chlorine for different periods of time. (b) Hole concentration and hole mobility of CuSCN thin films exposed to Cl_2_ for different periods of time. (c) Electronic DOS for pristine CuSCN (black line) and for CuSCN with a Cl interstitial impurity (red line) in the configuration shown in the inset. Zero energy is set at the highest occupied level, VB and CB refer to the valence band and the conduction band, respectively (colors used in Inset: Cu: brown, S: yellow, C: gray, N: blue, Cl: green spheres). (d) Transmittance and absorbance spectra of CuSCN/UV-graded quartz exposed to Cl_2_ for different periods of time.

According to the work done by Perera et al.,^37^ exposure of CuSCN to chlorine yields SCN^–^ ions, increases the carrier concentration in CuSCN layers, and then improves the conductivity of CuSCN thin film. In addition, as shown by the DFT calculations reported in Figure 1c, Cl interstitial species dope the system with holes by appropriately adjusting its electronic DOS profile. Therefore, the *I*–*V* curve measurements were performed to compare the electrical properties of CuSCN thin films before and after exposure to Cl_2_. The current flowing through the CuSCN thin film was increased by five orders of magnitude compared to that through pristine CuSCN thin films, indicating that exposure to Cl_2_ improved the conductivity of CuSCN thin films. Moreover, the current depends on the duration of exposure to Cl_2_ and reaches a maximum at an exposure time of 15 min. However, because chlorine is a corrosive gas, exposure for more than 15 min reduced the conductivity and decreased the current flowing through the thin films, as shown in Figure 1a. Because different exposure times resulted in different carrier concentrations, we could make CuSCN with the desired carrier concentration by controlling the exposure time.

To understand the carrier type and concentrations of Cl_2_-infused CuSCN, we performed Hall effect measurements, as shown in Figure 1b. The measured characteristics suggest the carrier type is hole-dominant and the hole concentration depends on the exposure time to chlorine. The maximum hole concentration was acquired for an exposure time of 15 min and slightly decreased when the exposure time was increased over 15 min in agreement with the *I*–*V* curve measurements in Figure 1a. For an inorganic semiconductor material, the concentration of holes is usually inversely correlated with hole mobility due to the carrier–carrier scattering, meaning that the higher the concentration of holes is, the lower is hole mobility.^48^ In the case of CuSCN, there are no carbon–carbon bonds or carbon–hydrogen bonds in the CuSCN molecules. Therefore, these molecules are considered to be inorganic materials, indicating that the high hole concentration leads to low hole mobility. According to the Hall effect measurements of chlorine-infused CuSCN thin films (Figure 1b), the concentration of holes is inversely correlated with hole mobility, which is based on the fact that CuSCN is an inorganic material.

DFT results on the effect of Cl impurities on the electronic properties of CuSCN are consistent with the experimental observations about an enhancement of hole conductance after Cl infusion. In particular, DFT calculations have identified a number of possible configurations for a Cl interstitial impurity inside the bulk of CuSCN. In the most stable configuration shown in the inset of Figure 1c, the Cl atom is two-fold coordinated to two proximal Cu atoms of CuSCN. As shown in the corresponding DOS plot, this Cl impurity shifts the Fermi level of the material inside the valence band and close to its maximum, so it results in an increase of the hole concentration, as observed. Interestingly, the most stable configuration for a Cl adatom on the (101̅0) surface of CuSCN has a similar effect, namely, it enhances the p-type character of the host by placing the Fermi level inside the valence band of the material.

In an attempt to understand if exposure to chlorine influences the optical properties of CuSCN thin films, we performed transmittance and absorbance measurements on 250 nm-thick CuSCN films coated on UV-graded quartzes. The transmittance and absorbance spectra of CuSCN thin films on UV-graded quartzes were not affected by exposure to chlorine, even after the longest exposure time (25 min), which is beneficial for the application of CuSCN thin films in wide band gap optoelectronic devices, as shown in Figure 1d.

**Figure 2:**
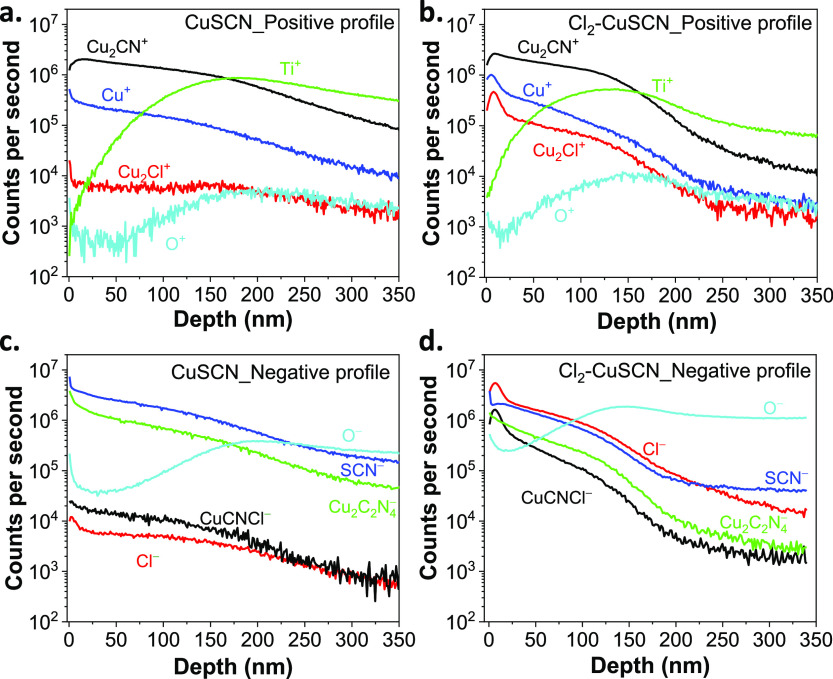
Secondary ion mass spectra of intrinsic CuSCN thin films and Cl_2_-infused CuSCN thin films. Positive SIMS profiles of (a) intrinsic CuSCN and (b) Cl_2_-infused CuSCN thin films. Negative SIMS profiles of (c) intrinsic CuSCN and (d) Cl_2_-infused CuSCN thin films.

In order to explore the incorporation of chlorine into CuSCN films, we conducted depth-profiling experiments using a SIMS technique (Figure 2). Both intrinsic CuSCN and Cl_2_-infused CuSCN films were analyzed, and the data were collected in both positive and negative modes. To acquire positive depth profiling data, the intensities of the selected positive ions assigned to Cu^+^, O^+^, Ti^+^, Cu_2_Cl^+^, and Cu_2_CN^+^ were measured as a function of the sputtering depth (Figure 2a for CuSCN and Figure 2b for Cl_2_–CuSCN). Similarly, negative depth profiling data were obtained by measuring the intensity of the selected negative ions assigned to SCN^–^, O^–^, Cl^–^, CuCNCl^–^, and Cu_2_C_2_N_4_^–^ as a function of the sputtering depth (Figure 2c for CuSCN and Figure 2d for Cl_2_–CuSCN). The SIMS data confirmed that chlorine was successfully incorporated into the CuSCN film, as evident by the significant increase in the Cl-containing signals (Cl^–^, CuCNCl^–^, and Cu_2_Cl^+^) for the Cl_2_–CuSCN film, compared to the intrinsic CuSCN film, for which the Cl signal (mainly due to Cl common contamination) was two orders of magnitude lower. Moreover, the Cl signal was detected throughout the entire depth of the film and exhibited the highest intensity in the first 10 nm, suggesting chlorine accumulated close to the surface. Finally, the slow decay trend of ion signals assigned to the film together with the slow rise of the signals assigned to the substrate (mainly O^–^ and Ti^+^) are very likely due to the pronounced roughness of the prepared film.

**Figure 3:**
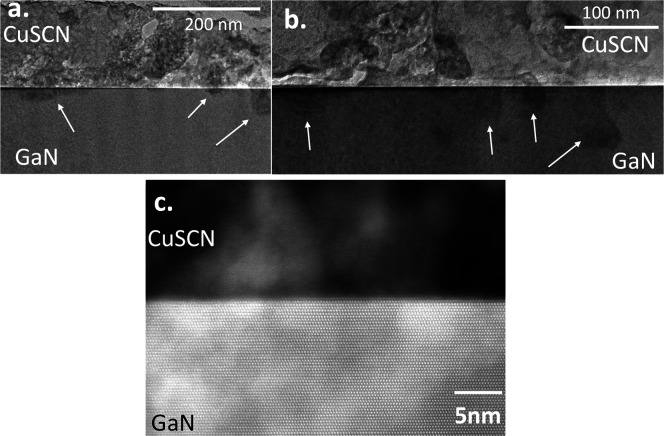
TEM and HR-STEM images of the interface of a p-CuSCN/n-GaN heterojunction. (a) Bright-field TEM image of the interface of a p-CuSCN/n-GaN heterojunction. (b) Bright-field TEM image of another cross-section of the interface of a p-CuSCN/n-GaN heterojunction. The areas indicated by white arrows are diffusion spots. (c) Atom-revealing HR-STEM image of the interface of a p-CuSCN/n-GaN heterojunction.

The exceptional properties of p-CuSCN encouraged us to combine this material with n-GaN to form a novel hybrid interface. We used transmission electron microscopy (TEM) and high-resolution scanning TEM (HR-STEM) to investigate the interface of p-CuSCN and n-GaN (see in Figure 3). In the cross section of the p-CuSCN/n-GaN structure, we observed some darker spots (indicated by the white arrows Figure 3a,b) in the n-GaN layer, which indicates elements from the CuSCN layer diffused into the GaN layer. HR-STEM image of the area of diffusion unveiled the irregular morphology of the spots, as shown in Figure 3c. The irregularity and random distribution of the spots might have occurred due to the amorphousness of the solution-processed CuSCN thin film.

**Figure 4:**
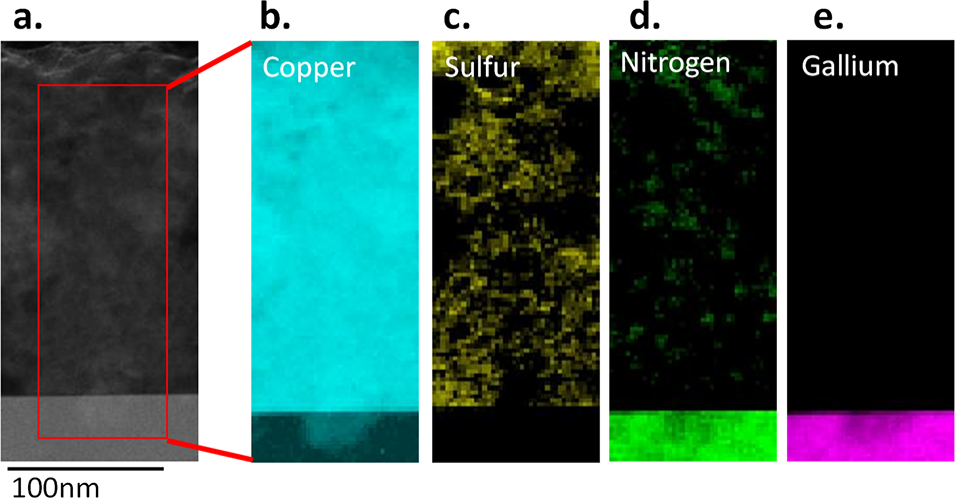
HAADF–STEM images and STEM–EELS measurements of the p-CuSCN/n-GaN structure. (a) HAADF–STEM image of a p-CuSCN/n-GaN structure. The area within the red line indicates the region of STEM–EELS scanning. (b) Copper signal from STEM–EELS measurements. (c) Sulfur signal from STEM–EELS measurements. (d) Nitrogen signal from STEM–EELS measurements. (e) Gallium signal from STEM–EELS measurements.

We then performed high-angle annular dark field (HAADF)–STEM imaging combined with electron energy loss spectroscopy (EELS) to investigate the elemental components in the diffusion area. In the HAADF–STEM images, we included the possible diffusion area located at the interface of the CuSCN layer and the GaN layer during the STEM–EELS scanning to obtain a comprehensive view of the elemental distributions, as shown in Figure 4a. The STEM–EELS measurements shown in Figure 4b–d revealed Cu, S, and N signals in the CuSCN layer and Ga and N signals in the GaN layer, as expected. Moreover, copper signals were detected in the CuSCN layer and GaN layer, which suggests that the element diffusing into the GaN layer is copper, as shown in Figure 4b. Consolidation of the STEM–EELS images of copper, gallium, and nitrogen revealed a copper signal in the GaN region located in the regions where the gallium and nitrogen signals were lowest, which indicates that copper blocked gallium and nitrogen during the diffusion process.

High-resolution XPS measurements were employed to determine the valence band offset (VBO) of the CuSCN/GaN heterojunction interface. To evaluate the VBO at the GaN/CuSCN heterointerface, the energy difference between the Ga 2p_3/2_ and Cu 2p_3/2_ core levels from the GaN/CuSCN heterojunction sample and the energy of Ga 2p_3/2_ and Cu 2p_3/2_ core levels relative to the respective valence band maximum (VBM) of the GaN and CuSCN samples, respectively, need to be acquired.

The VBO for the GaN/CuSCN heterojunction can be calculated by the method provided by Kraut et al.,^49^ which is expressed as
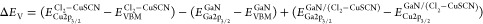
1
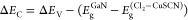
2

**Figure 5:**
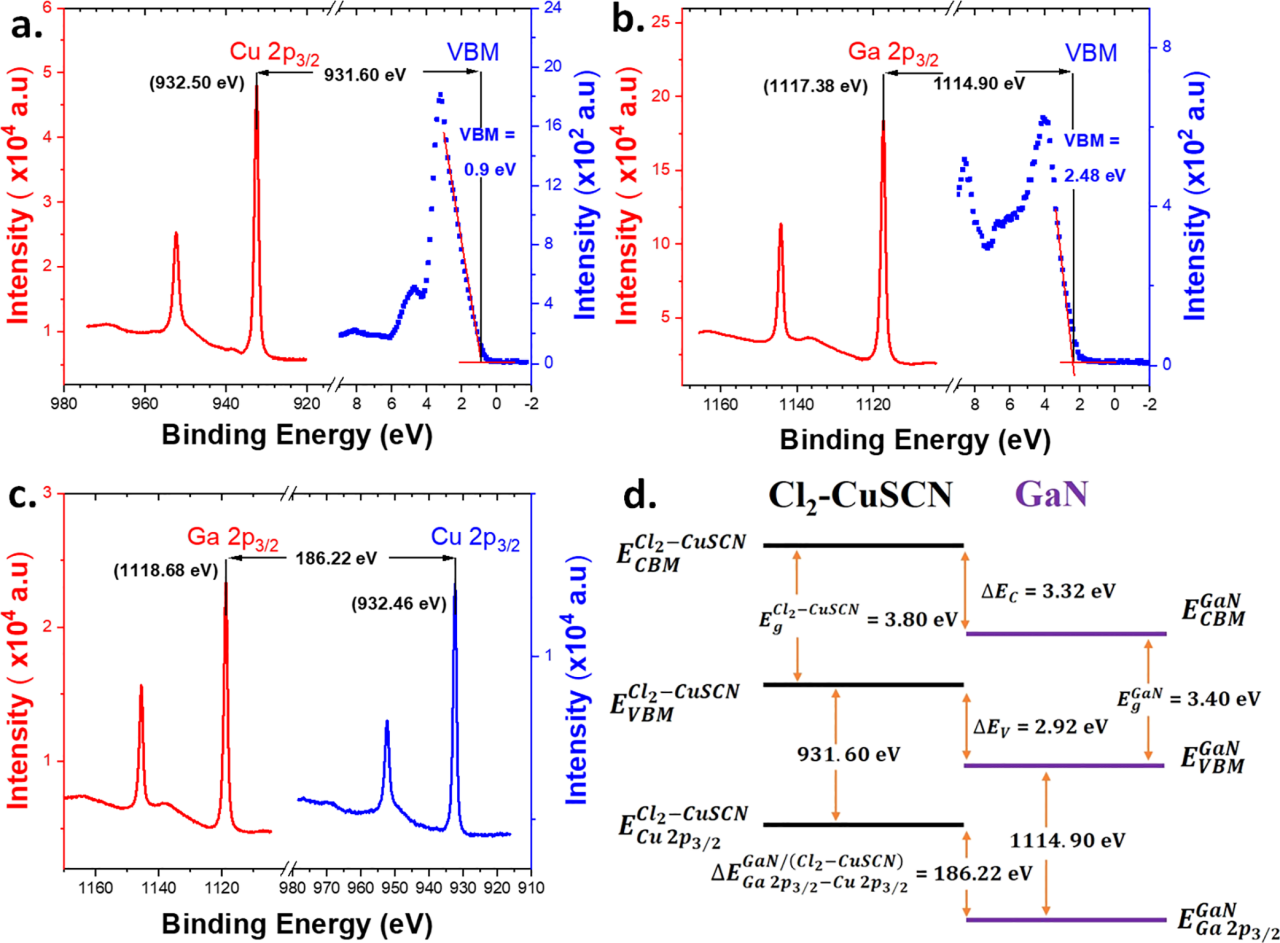
High-resolution XPS measurements. (a) Cu 2p core-level and valence band spectrum for CuSCN. (b) Ga 2p core-level and valence band spectrum for GaN. (c) Ga 2p and Cu 2p core-levels for the GaN/CuSCN heterojunction. (d) Schematic representation of band alignment at the GaN/CuSCN heterointerface.

Figure 5a shows the Cu 2p core-level and valence band spectra of the bulk CuSCN sample. The binding energy of Cu 2p_3/2_ is equal to 932.50 eV and the VBM is equal to 0.9 eV. The separation between the core-level energy of Cu 2p_3/2_ and the VBM, , for CuSCN was determined to be 931.60 eV.

Figure 5b shows the Ga 2p core level and valence band spectra of the bulk GaN sample. The binding energy of Ga 2p_3/2_ is equal to 1117.38 eV, and the VBM is equal to 2.48 eV. The separation between the core-level energy of Ga 2p_3/2_ and the VBM, , for GaN was determined to be 1114.90 eV.

Figure 5c shows the Ga 2p and Cu 2p core-level spectra of the thin CuSCN film grown on GaN. The binding energies of Ga 2p_3/2_ and Cu 2p_3/2_ are 1118.68 and 932.46 eV, respectively. The energy difference, 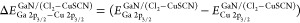 between the Ga 2p_3/2_ and Cu 2p_3/2_ core levels was observed to be 186.22 eV.

After substitution in eq 1, the VBO was found to be Δ*E*_v_ = 2.92 eV. The optical band gaps of GaN and CuSCN are *E*_g_^GaN^ = 3.40 eV and *E*_g_^CuSCN^ = 3.80 eV, respectively. Thereby, substitution of the VBO (Δ*E*_v_) obtained from XPS analysis and the electronic band gap (*E*_g_) values of GaN and CuSCN in eq 2 allowed us to determine the conduction band offset (CBO) Δ*E*_C_ for the GaN/CuSCN heterojunction. Hence, the measured CBO (Δ*E*_C_) is 3.32 eV. The offset parameters determined in this study are represented in the schematic band alignment diagram in Figure 5d, which shows that the band alignment pertains to a type-II heterojunction.

**Figure 6:**
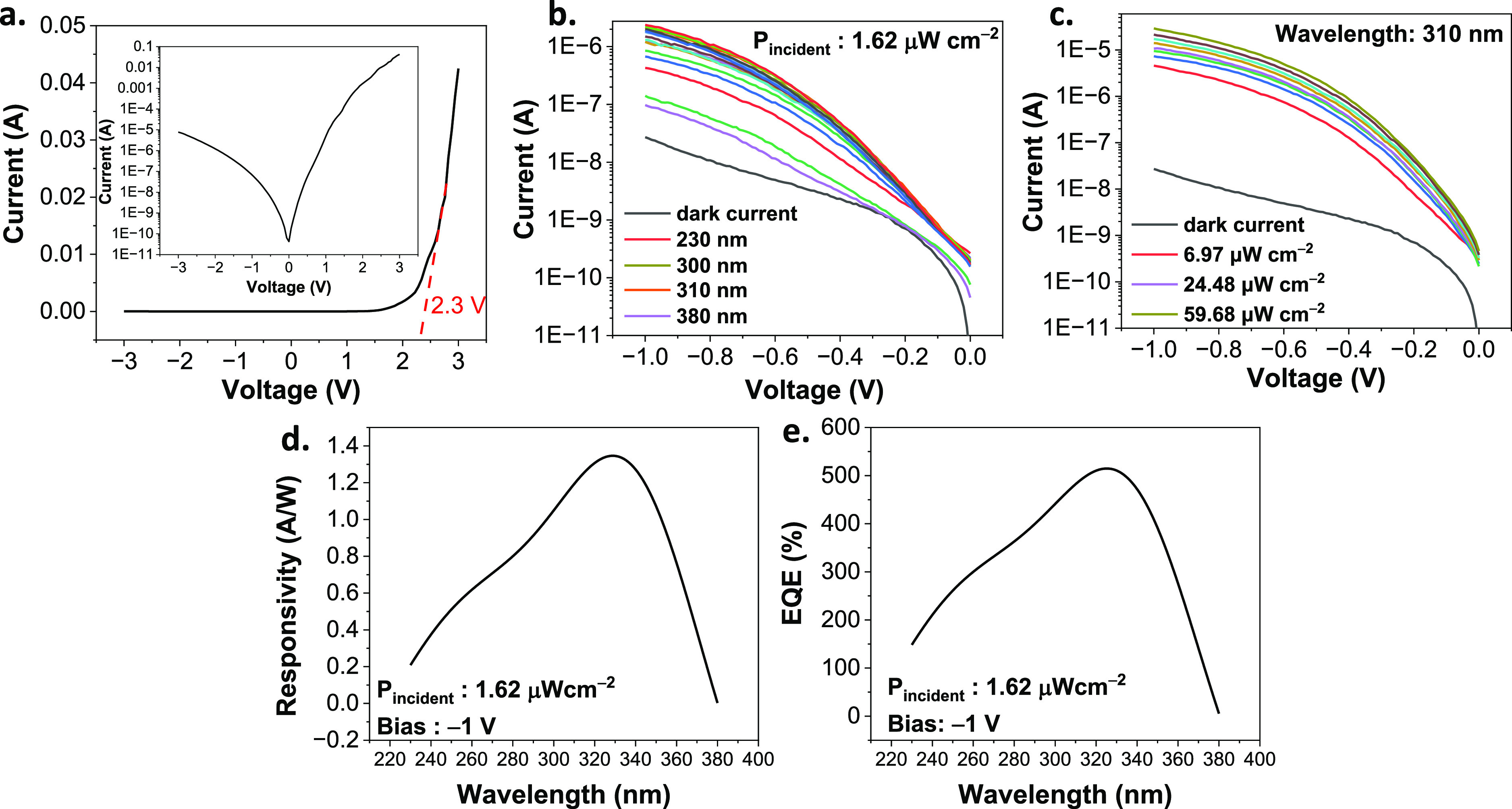
Device characterization of the as-fabricated p-CuSCN/n-GaN UVPD. (a) *I*–*V* curve sweeps from −3 to 3 V. The inset plot shows the *I*–*V* curve in log-scale. (b) Photocurrent measurements of the p-CuSCN/n-GaN photodetector for incident wavelengths ranging from 230 to 380 nm. (c) Power-dependent photocurrent measurements of the p-CuSCN/n-GaN device. The incident wavelength is 310 nm. (d) Plots of responsivity versus wavelength at a reverse bias of 1 V. (e) Plots of EQE versus wavelength at an input power of 1.62 μW/cm^2^ and reverse bias of 1 V.

We performed several experiments to understand the basic performance of the device, including the current–voltage characteristics and photoelectrical measurements. The *I*–*V* curve revealed that the turn-on voltage of the *p*-CuSCN/n-GaN heterojunction is 2.3 V, and the inset plot with the *y*-axis in the log scale demonstrated that the current flow is four orders of magnitude higher at forward bias than in reverse bias, indicating the formation of a p-CuSCN/n-GaN semiconductor junction (see Figure 6a). Depending on the band alignment, the Δ*E*_c_ and Δ*E*_v_ are 3.32 and 2.92 eV, respectively, indicating that the turn-on voltage of the corresponding device should be around 3.3 V. However, the measured turn-on voltage for the p-CuSCN/n-GaN device was 2.3 V. Thus, the values predicted based on the band alignment are not in agreement with the turn-on voltage of the practical devices. The TEM images in Figure 3 show that the copper ions diffusing into the GaN layer formed connections with those in the CuSCN layer. These connections functioned like the roots of a plant to further increase the area of contact between the p-CuSCN layer and the n-GaN layer and create leakage paths. According to work by Dobos et al.,^50^ the barrier between layers is inversely correlated with the area of contact between them. The larger the area of contact is, the lower is the barrier between them. Consequently, we think that the diffusion of copper into the GaN layer increased the area of contact between the p-CuSCN and the n-GaN layers and created leakage paths to reduce the turn-on voltage of the as-fabricated p-CuSCN/n-GaN UVPD. Therefore, even though the band alignment indicates the turn-on voltage of p-CuSCN/n-GaN is around 3.3 V, the turn-on voltage of the actual device was 2.3 V.

An arc lamp was used as the light source to measure the responsivity of the as-fabricated p-CuSCN/n-GaN device at particular wavelengths. Because the span of wavelengths exhibits different power levels, ND filters were used to calibrate the power to 1.62 μW cm^–2^. Based on measured features, the responsivity with respect to the illumination wavelength can be calculated by the following formula
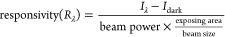
3

According to the mask design and optical setup, the exposed area of the devices is 1.02 × 10^–3^ cm^2^ and the rectangular beam size is 7.20 × 10^–2^ cm^2^, *I*_dark_ is the dark current, and *I*_λ_ is the photocurrent at a specific wavelength. The responsivity plot is shown in Figure 6d. The 230 nm wavelength led to a relatively low responsivity. As the wavelength of the incident light was increased, the responsivity became higher, reaching a maximum of 1.35 A/W under illumination with a peak wavelength of 330 nm. Moreover, the responsivity was maintained above 1.0 A/W in the range from 300 to 360 nm, which indicates that GaN contributes to the absorption of light, and the CuSCN thin film results in high responsivity. Because the CuSCN layer in this first report of a prototypical p-CuSCN/n-GaN heterojunction-based UV-light photodetector is from a solution-processed method that yields many traps in the thin film, photocurrent flowing through the CuSCN layer extracts trapped carriers in the traps. This increases the photocurrent and then yields a high responsivity.^22,23^ Based on the measured characteristics of the as-fabricated p-CuSCN/n-GaN photodetectors, we think that traps in the CuSCN layer contribute to the extraordinary responsivity above 1 A/W in the range of wavelengths from 300 to 350 nm. In the power-dependent photocurrent measurements, the higher the power of the 310 nm incident wavelength, the stronger the photocurrent generated by p-CuSCN/n-GaN, which confirms that the device was responding to the power of the light accordingly, as shown in Figure 6c. Based on the responsivity shown in Figure 6d, the EQE (η_λ_) can be calculated as
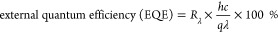
4where *h* is Planck’s constant, *c* is the speed of light, *q* is the elementary charge, and λ is the radiation wavelength. The corresponding EQE of the p-CuSCN/n-GaN photodetector measured at a voltage bias of −1 is shown in Figure 6e. Because the expression for the EQE contains the term representing responsivity, traps from the solution-processed CuSCN thin film also increase the calculated EQE values to beyond 100%, indicating that multiple electrons were generated by the photocurrent in the p-CuSCN/n-GaN photodetectors. The peak EQE of 5.14 × 10^2^% was observed at a wavelength of 330 nm. The EQE values drop once the incident wavelength is outside the range of 300–360 nm, consistent with the trend in responsivity. Compared to other III-nitride-based UVPDs, the p-CuSCN/n-GaN heterojunction UVPD has higher responsivity and EQE, which benefit the device operation under low power incident light. However, even though the responsivity of p-CuSCN/n-GaN UVPD is not as high as that of a CuSCN M-S-M UVPD, the p-CuSCN/n-GaN device has a better response time, as shown in Table 1.

**Table 1:** Comparison of III-Nitride-Based and CuSCN-Based UVPDs

structure	turn-on voltage (v)	responsivity (A/W)	EQE (%)	rise time/fall time (second)	refs
CuSCN MSM	0.6	79 (280 nm)	N/A	1.0/∼10	(22)
AlGaN p–i–n	N/A	0.1 (315 nm)	39 (315 nm)	N/A	(51)
AlN nanowire MSM	N/A	6 × 10^–4^ (200 nm)	N/A	1.3/2.0	(52)
p-CuSCN/n-GaN	2.3	1.35 (330 nm)	514 (330 nm)	6.25 × 10^–4^/1.3 × 10^–3^	this work

**Figure 7:**
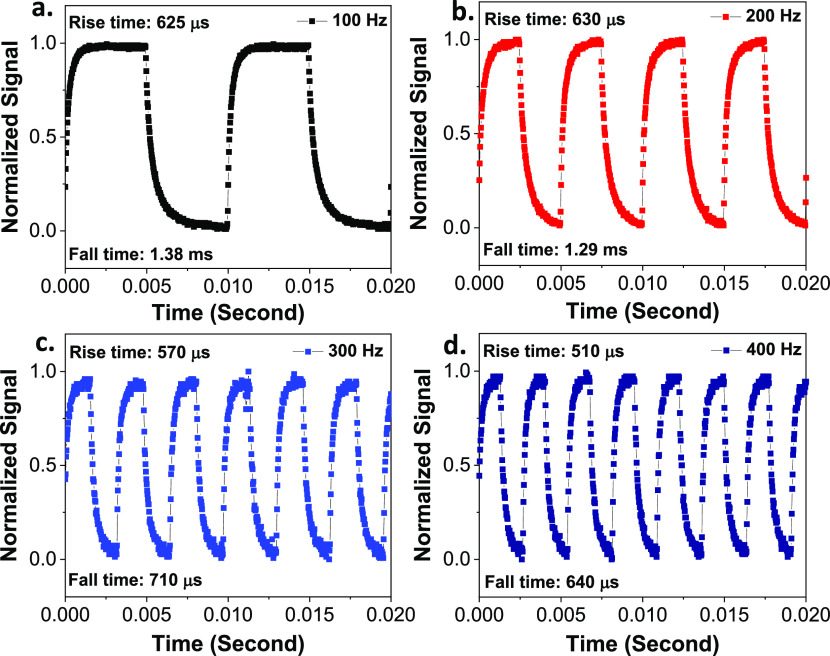
Time response tests of the as-fabricated p-CuSCN/n-GaN photodetector under illumination with a 266 nm laser with an optical chopper at 0 bias. (a) Chopper frequency set to 100 Hz. The rise time and fall time of the PD are 625 μs and 1.38 ms, respectively (b) Chopper frequency set to 200 Hz. The rise time and fall time of the PD are 630 μs and 1.29 ms, respectively. (c) Chopper frequency set to 300 Hz. The rise time and fall time of the PD are 570 and 710 μs, respectively. (d) Chopper frequency set to 400 Hz. The rise time and fall time of the PD are 510 and 640 μs, respectively.

Figure 7 presents the time response tests of the p-CuSCN/n-GaN UVPD performed under illumination with a 266 nm laser at different chopper frequencies. At 100 and 200 Hz, the rise and fall times are basically around 600 μs and 1.3 ms, respectively, which indicates that the UVPD has a slow response speed. When the chopper frequency was increased beyond 200 Hz, the rise and fall times reduced to 570 and 710 μs at 300 Hz and 510 μs and 640 μs at 400 Hz, respectively. The reduction in the response time indicates that the frequency of the signal is approaching the time response limit of the device.

## Conclusions

In this work, the hole transport properties of wide-band gap CuSCN films were enhanced by exposure to Cl_2_ gas using a dry etching system for the first time. Optical characterization showed that Cl_2_ treatment does not influence the absorption spectra of the CuSCN layers. However, electrical characterization of the resulting p-CuSCN films revealed a direct correlation between the hole concentration and the duration of exposure to Cl_2_ gas. SIMS analysis indicated that Cl_2_ diffuses across the entire CuSCN layer, and hence uniformly impacts the electrical properties of the layer. XPS measurements provided information on the band alignment between the solution-processed p-CuSCN and n-GaN and revealed that the VBO is 2.92 eV and CBO is 3.32 eV. Based on the type-II band alignment which was verified for the first time, a prototypical p-CuSCN/n-GaN heterojunction UVPD was fabricated and characterized. The photodetector exhibited a turn-on voltage of 2.3 V, which was lower than the CBO calculated from the band alignment analysis. TEM analysis of the p-CuSCN/n-GaN interface revealed that copper diffuses into the GaN layer; we suggest that this explains the reduced turn-on voltage. The p-CuSCN/n-GaN photodetectors showed a responsivity of 1.35 A/W and EQE of 5.14 × 10^2^% under illumination at a wavelength of 330 nm and a reverse bias of −1 V. Finally, analysis of the time response yielded rise and fall times of over 500 μs. Our findings are significant, as a plethora of hybrid optoelectronic devices of inorganic and organic natures can be realized based on Cl_2_-infused CuSCN as an alternative hole-injection layer.

## Supporting Information Available

Fabrication process for the p-CuSCN/n-GaN heterojunction UVPD (PDF)
