# Supplementary material for: Chlorine-Infused Wide-Band Gap p-CuSCN/n-GaN Heterojunction Ultraviolet-Light Photodetectors
Source: ACS Appl Mater Interfaces. 2022 Apr 11;14(15):17889–98. doi: 10.1021/acsami.1c22075 (PMC9026266; doi:10.1021/acsami.1c22075)
Supplement: Supplementary file 1 — am1c22075_si_001.pdf [file am1c22075_si_001.pdf]

Supporting Information

Chlorine-infused wide-bandgap p-CuSCN/n-GaN heterojunction ultraviolet-light photodetectors

Jian-Wei Liang,[1,†] Yuliar Firdaus,[2,4,†] Chun Hong Kang,[1] Jung-Wook Min,[1] Jung-Hong Min,[1] Redha H. Al Ibrahim,[1] Nimer Wehbe,[3] Mohamed Nejib Hedhili,[3] Dimitrios Kaltsas,[5] Leonidas Tsetseris,[5] Sergei Lopatin,[3] Shuiqin Zheng,[1] Tien Khee Ng,[1] Thomas D. Anthopoulos,[2,] and Boon S. Ooi.[1,*]*

[1]Photonics Laboratory, Computer, Electrical and Mathematical Sciences and Engineering Division (CEMSE), King Abdullah University of Science and Technology (KAUST), Thuwal, 23955-6900, Saudi Arabia

[2]Physical Science and Engineering Division (PSE), KAUST Solar Center (KSC), King Abdullah University of Science and Technology (KAUST), Thuwal, 23955-6900, Saudi Arabia.

[3]Imaging and Characterization Core Labs, King Abdullah University of Science and Technology (KAUST), Thuwal, 23955-6900, Saudi Arabia.

[4]Currently with Research Center for Electronics and Telecommunication, National Research and Innovation Agency, Jalan Sangkuriang Komplek LIPI Building 20 Level 4, Bandung 40135, Indonesia.

[5]Department of Physics, School of Applied Mathematical and Physical Sciences, National Technical University of Athens, GR-15780 Athens, Greece.

[†]Authors contributed equally

Corresponding Authors

[*]Authors to whom correspondence should be addressed: thomas.anthopoulos@kaust.edu.sa, boon.ooi@kaust.edu.sa

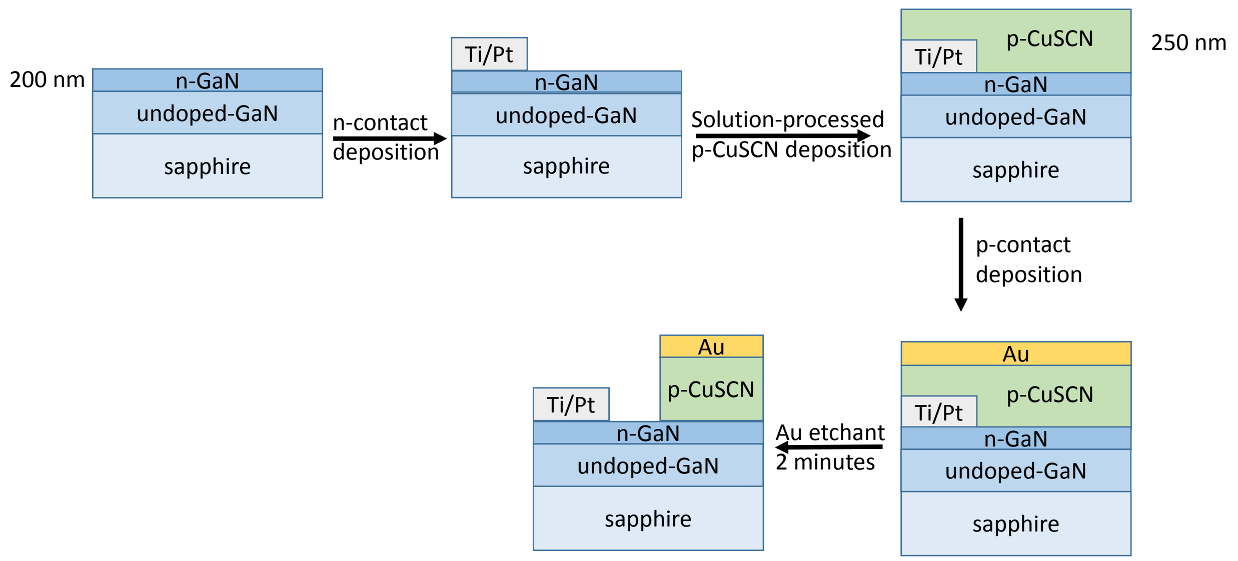

Figure S1. Fabrication process for the p-CuSCN/n-GaN heterojunction ultraviolet-based photodetector.

Pt (50 nm)/Ti (50 nm) metal contacts were deposited on n-GaN through a lift-off process, and then we annealed the samples at 600 ˚C for 2 minutes. To deposit the CuSCN thin film layer, 60 mg of CuSCN powder (Sigma-Aldrich) was dissolved in 1 mL diethyl sulfide, filtered through a polymer filter, and spin-coated onto the as-fabricated n-GaN/sapphire template at 1500 rpm for 30 seconds. The CuSCN-coated n-GaN/i-GaN/sapphire sample was placed on a hot plate at 105 ˚C for 10 min to remove the solvent. The thickness of the as-coated CuSCN layer was 250 nm. Next, the CuSCN thin film was exposed to chlorine using a dry etching system consisting of a vacuum chamber with controllable gas valves and tunable chamber pressure, then Au thin film (175 nm) was deposited

onto the CuSCN thin film by magnetron sputtering. In order to etch away the Au/CuSCN bilayer that was blocking the Pt/Ti contact, an AZ 5214E photoresist was spin-coated onto the Au/CuSCN bilayer at 3000 rpm for 30 seconds, patterned using a laser writer, and developed in developer solution. Once the protection layer was ready, gold etchant was used to remove both the Au layer and CuSCN layer, then the photoresist layer was removed by acetone.
